# Inhibitors of ABCB1 and ABCG2 overcame resistance to topoisomerase inhibitors in small cell lung cancer

**DOI:** 10.1111/1759-7714.14527

**Published:** 2022-06-20

**Authors:** Miwako Omori, Rintaro Noro, Masahiro Seike, Kuniko Matsuda, Mariko Hirao, Aya Fukuizumi, Natsuki Takano, Akihiko Miyanaga, Akihiko Gemma

**Affiliations:** ^1^ Department of Pulmonary Medicine and Oncology Graduate School of Medicine, Nippon Medical School Tokyo Japan

**Keywords:** ATP‐binding cassette transporters, drug resistance, small cell lung cancer, topoisomerase I inhibitor, topoisomerase II inhibitor

## Abstract

**Background:**

Small cell lung cancer (SCLC) is a highly aggressive disease with a poor prognosis. Although most patients initially respond to topoisomerase inhibitors, resistance rapidly emerges. The aim, therefore, is to overcome resistance to topoisomerase I (irinotecan) or II (etoposide) inhibitors in SCLCs.

**Methods:**

To identify key factors in the chemoresistance of SCLCs, we established four cell lines resistant to etoposide or an active metabolite of irinotecan, SN‐38, from SCLC cell lines and evaluated RNA profiles using parental and newly established cell lines.

**Results:**

We found that the drug efflux protein, ATP‐binding cassette sub‐family B member 1 (ABCB1), was associated with resistance to etoposide, and ATP‐binding cassette sub‐family G member 2 (ABCG2) was associated with resistance to SN‐38 by RNA sequencing. The inhibition of ABCB1 or ABCG2 in each resistant cell line induced synergistic apoptotic activity and promoted drug sensitivity in resistant SCLC cells. The ABC transporter inhibitors, elacridar and tariquidar, restored sensitivity to etoposide or SN‐38 in in vitro and in vivo studies, and promoted apoptotic activity and G2‐M arrest in resistant SCLC cells.

**Conclusions:**

ABC transporter inhibitors may be a promising therapeutic strategy for the purpose of overcoming resistance to topoisomerase inhibitors in patients with SCLC.

## Graphical abstract

RNA sequencing of cancer cells in this study revealed ABCB1 and ABCG2 to associate with resistance to topoisomerase inhibitors. This study suggests that ABC transporter inhibitors may be a promising therapeutic strategy for the purpose of overcoming resistance to topoisomerase inhibitors in patients with small‐cell lung cancer.

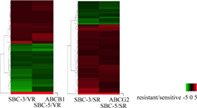

## INTRODUCTION

Small cell lung cancer (SCLC) is a highly aggressive disease with a 5‐year survival rate of less than 7%.^1^ Patients are often diagnosed with extensive disease. Chemotherapy has a major role in the treatment of patients with advanced SCLC because of few molecular target therapies. Cisplatin combined with a topoisomerase inhibitor, such as etoposide or irinotecan, is standard chemotherapy for SCLC. DNA topoisomerases are enzymes that induce DNA strand recombination and cell proliferation following DNA strand breakes.^2^ Type I and II enzymes cleave one and both DNA strands, respectively. Topoisomerase inhibitors suppress enzyme activities and inhibit cancer cell proliferation. Etoposide is a type II inhibitor and irinotecan is a type I inhibitor. Most patients respond initially to chemotherapy, but resistance rapidly emerges.^3^ Recently, the IMpower133 study and CASPIAN phase III trials showed an overall survival benefit for atezolizumab and durvalumab combined with standard platinum‐etoposide chemotherapy in a first‐line SCLC setting.^4^, ^5^, ^6^, ^7^ However, chemotherapy is still a key therapy for SCLC even with the availability of combined immunotherapy. Some SCLCs are intrinsically resistant to chemotherapy and in virtually all cases even initial responders rapidly develop acquired resistance. A therapeutic strategy to overcome chemoresistance is therefore required.

The resistance mechanisms of SCLC are unknown. Unlike for non‐small‐cell lung cancer (NSCLC), a patient with recurrent SCLC is rarely given a second biopsy because of rapid progression, making research on the mechanism of resistance using clinical specimens difficult.^8^ In this study, we established topoisomerase inhibitor‐resistant cells of SCLC and analyzed differences between resistant and sensitive cells to clarify the mechanism(s) involved in overcoming resistance to topoisomerase inhibitors.

## METHODS

### Cell culture

We used 12 SCLC cell lines: SBC‐3 and SBC‐5 from the Japanese Collection of Research Bioresources Cell Bank, H69, H69AR, H719, H1048, H1105, H1417, DMS53, and H1882 from the American Type Culture Collection, and MS‐1 and Lu‐139 from the Riken Cell Bank. SBC‐3 and SBC‐5 were cultured in MEM‐EAGLE medium (Sigma‐Aldrich) with 10% fetal bovine serum (FBS; Biowest) and 1% penicillin/streptomycin (Fujifilm Wako). H69AR was maintained in RPMI‐1640 (Fujifilm) with 20% FBS and 1% penicillin/streptomycin. The other SCLC cell lines were maintained in RPMI‐1640 with 10% FBS and 1% penicillin/streptomycin. These cell lines were obtained between 2008 and 2017 and were routinely examined for the absence of mycoplasma.

### Drugs and growth inhibition assay

Etoposide, SN‐38, and tariquidar were purchased from Selleck Chemicals (Houston). Elacridar was purchased from Santa Cruz Biotechnologies. Topotecan was purchased from Cayman Chemical. Cisplatin was purchased from Fujifilm Wako. The effects of etoposide and SN‐38 on SCLC cell lines were assessed by tetrazolium (MTS) assay as previously described.^9^, ^10^, ^11^, ^12^ Cell suspensions (5000 or 10 000 cells/well) were seeded into 96‐well plates and increasing concentrations of drugs or vehicle (dimethyl sulfoxide) added. After incubation at 37°C for 72 h, 10 μl of Cell Counting Kit‐8 (Dojindo) was added to each well and cells further incubated at 37°C for 90 min. After shaking the plate, absorbances were measured at a test wavelength of 450 nm using a microplate reader (Infinite M200 PRO; Tecan Group Ltd). The IC50 value was defined as the concentration of etoposide or SN‐38 needed for a 50% reduction in growth. All experiments were independently repeated more than three times.

### RNA isolation and sequencing

Total RNA was isolated from SCLC cell lines by TRIzol reagent (Thermo Fisher Scientific) as previously described.^11^, ^13^ The quantity of total RNA was determined using a NanoDrop 2000 Spectrophotometer (Thermo Scientific). RNA sequencing of extracted RNA was conducted on a HiSeq 2500 platform using paired‐end reads (Illumina). Reads were aligned against a reference human genome and compared with each cell line. Total RNA (10–100 ng) was used to construct libraries using a TruSeq RNA Kit (Illumina). Libraries were used for the generation of clustered flow cells on cBot using a TruSeq PE Cluster Kit v2 (Illumina). Paired‐end sequencing (75‐bases) was performed on a Genome Analyzer IIx sequencer using a TruSeq SBS Kit v2 (Illumina). Illumina software was used for processing image data into raw sequencing data (CLC Biomedical Genomics Workbench; Qiagen).^14^ For RNA sequencing, we mapped RNA reads using BaseSpace App: RNA–Seq Alignment v2.0.1 (Illumina) with STAR and an hg38 reference^15^. RNA sequencing alignment data was analyzed by RNA‐Seq Differential Expression Ver 1.0.1 (Illumina). RNA sequencing read counts were normalized to transcripts per million for quantitative representation. We selected genes in a sensitive/resistant comparison on the basis of a fold change of >4. RNA sequencing data were deposited in a Sequence Read Archive (SRA) database using the accession number, PRJNA717912. Tumor RNA‐sequencing had an average of 9 956 750 aligned reads (range 5 942 630–15 876 755; Table S1). A custom bioinformatics pipeline was used to perform sequence alignment, variant calling, and variant filtering.

### Western blot

Protein samples of cells were lysed in buffer containing 50 mM Tris–HCl, pH 7.6, 150 mM NaCl, 0.1% sodium dodecyl sulfate, 1% Nonidet P‐40, and 0.5% sodium deoxycholate, and western blotting was performed as previously described.^9^, ^16^ Quantification was achieved by densitometry using ImageJ software (National Institutes of Health).

Membranes were incubated with the following antibodies: Antibodies against ATP‐binding cassette sub‐family G member 2 (ABCG2; #4477), phosphorylated‐Cdc2 Tyr15 (p‐Cdc2; #9111), phosphorylated‐Chk1 Ser345 (p‐Chk1; #2348), cleaved poly ADP ribose polymerase (cleaved PARP; #5625), topoisomerase I (TOP1; #79971), and topoisomerase IIα (TOP2a; #12286) were purchased from Cell Signaling Technology. Antibodies against ATP‐binding cassette sub‐family B member 1 (ABCB1; #sc55510) and glyceraldehyde 3‐phosphate dehydrogenase (GAPDH; #sc47724) were purchased from Santa Cruz Biotechnology.

### Real‐time quantitative reverse transcription‐polymerase chain reaction

*TOP1* and *TOP2a* gene expressions were assessed by real‐time quantititive reverse transcription (qRT) ‐PCR using the TaqMan Gene Expression Assay (Thermo Fisher Scientific). The cDNA was utilized using THUNDERBIRD SYBR qPCR/RT Set III (Toyobo) according to the manufacture's instructions. Gene expression levels were calculated using the 2‐ΔΔCt method.

### Oligonucleotide transfection

Small interfering RNAs (siRNAs) targeting ABCB1, ABCG2, and negative controls were purchased from Ambion: ABCB1 (ID: s10420), ABCG2 (ID: s18057), homologous negative controls (#4390844). After cell seeding, siRNAs of *ABCB1* and *ABCG2* were transfected using Lipofectamine 2000 reagent according to the manufacturer's instructions (Life Technologies). Small interfering RNA complexes were transfected into cells at a final concentration of 40 nM. The transfection medium was replaced 24 h later and cells were then incubated at 37°C for 48 h, with or without drugs.

### Colony formation assay

Cells were seeded (1 × 10^4^ cells/well) in six‐well plates with transfections of siRNA complexes at a final concentration of 40 nM and incubated overnight. The medium was changed and cells were incubated with 4 μM etoposide or 0.04 μM SN‐38 for 10–14 days at 37°C. Colonies were stained with 10% Microscopy Giemsa's azur eosin methylene blue solution (Merck) for 60 min at room temperature after washing with methanol and phosphate buffered saline (PBS), and colonies counted.

### Annexin V assay

Cells (4 × 10^5^ cells/well) were seeded onto six‐well plates in medium containing 5 μM etoposide or 0.05 μM SN‐38, with or without 2 μM elacridar or 1 μM tariquidar, and incubated at 37°C in 5% CO_2_. After 72 h of incubation, cells were trypsinized, collected, and stained with fluorescein isothiocyanate (FITC)‐conjugated annexin V and propidium iodide (PI) using an apoptosis detection kit (Nacalai Tesque Inc.) according to the manufacturer's protocol as previously prescribed.^17^ The cells were analyzed on a BD FACSVerse flow cytometer (10 000 events per sample; BD Bioscience). Fluorescence compensation and analysis were performed with Flow Jo software (BD Bioscience). The percentage of total apoptotic cells, which were both Annexin V positive and Annexin V/PI double positive cells, was calculated. Each experiment was performed independently three times.

### Cell cycle analysis

Cells were seeded (5 × 10^5^ cells/well) onto six‐well plates, with or without drugs, in medium containing 5 μM etoposide or 0.05 μM SN‐38, with or without 2 μM elacridar or 1 μM tariquidar, and incubated at 37°C in 5% CO_2_. After 24 h, cells were trypsinized, fixed, and stained using a FITC BrdU Flow Kit (BD Bioscience) according to the manufacturer's protocol. Before analysis, cells were incubated with 0.3 μg/μL of DNase A for 60 min at 37°C. Finally, cells were stained with 7‐aminoactinomysin D and washed in staining buffer (PBS, 3% FBS, and 2 mM EDTA). Stained cells were analyzed on a BD FACSVerse flow cytometer (10 000 events per sample). The percentage of cells in each cell cycle phase was determined using a cell‐cycle module within FlowJo software. Each experiment was performed independently three times.

### Drug efflux assay

Drug transport mediated by ABCB1 or ABCG2 was examined using a flow cytometry efflux assay as previously described.^18^, ^19^ Trypsinized cells were resuspended in medium containing 0.1 mg/mL rhodamine 123 or 5 μM mitoxantrone, in the presence or absence of 2 μM elacridar or 1 μM tariquidar, and incubated for 30 min at 37°C in 5% CO_2_. Cells were then washed, resuspended in staining buffer, and placed on ice until analyzed. Samples were analyzed by a BD FACSVerse flow cytometer (10 000 events per sample). Rhodamine 123 and mitoxantrone fluorescence were detected by a 488‐nm argon laser with 530‐nm bandpass filter, and a 633‐nm HeNe laser with 660‐nm bandpass filter, respectively. Data analysis was performed using FlowJo software. Right‐shifting of histograms of fluorescence intensity represented the inhibition of efflux through corresponding ABC transporters.

### Treatment of chemoresistant xenografts

Six‐week‐old male BALB/cAJcl *nu/ nu* mice were obtained from Clea Japan. SBC‐3/VR (1 × 10^7^) or SBC‐3/SR (1 × 10^7^) cells were injected subcutaneously in the flanks of mice. When tumors became measurable, mice were randomly assigned to the following three groups: vehicle; topoisomerase inhibitors etoposide or irinotecan (Nippon Kayaku), or topoisomerase inhibitors plus elacridar. Etoposide (8 mg/kg) was intraperitoneally administered to mice bearing SBC‐3/VR xenografts on days 1 to 3 weekly. Irinotecan (60 mg/kg) was intravenously administered to mice bearing SBC‐3/SR xenografts on days 1 to 3 weekly. Elacridar was orally administered at 40 mg/kg on days 1 to 5 weekly. The evaluation was estimated as the ratio of tumor growth compared with vehicle and etoposide or irinotecan alone in vivo. Tumor volume (*V*) was calculated using the following formula: *V* = length×(width)^2^/2. Experimental protocols were approved (approval number, 2020‐072) by the Animal Care and Use Committee of Nippon Medical School, Tokyo, Japan.

### Statistical analysis

A standard Student's *t*‐test was used to compare experimental data with a control group. All *p* values were two‐sided and the statistical significance was set at <0.05. Analyses were performed using SPSS statistical software, SPSS (ver. 25).

## RESULTS

### Identification of sensitive and resistant cell lines using the key anticancer drugs etoposide and SN‐38

**FIGURE 1:**
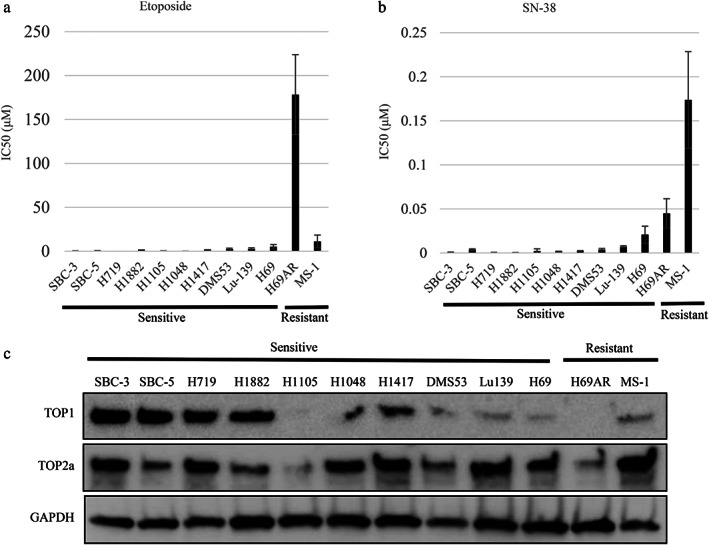
Half‐maximal inhibitory concentrations (IC50) for etoposide and SN‐38, and protein expression in small cell lung cancer (SCLC) cell lines. IC50 values for etoposide (a) and SN‐38 (b) in SCLC cells were determined by tetrazolium (MTS) assay to identify sensitive and resistant cells. There were no significant differences between sensitive and resistant cells (*p* = 0.46, 0.35, respectively). (c) Protein expression levels in SCLC cells were evaluated by western blot analysis. GAPDH, glyceraldehyde 3‐phosphate dehydrogenase; TOP, topoisomerase

We used two topoisomerase inhibitors, etoposide and SN‐38, an active metabolite of irinotecan, and evaluated their anticancer effect on 12 SCLC cell lines by MTS assay. We identified 10 sensitive and two resistant cell lines according to their half‐maximal inhibitory concentrations (IC50; Figure 1(a),(b)). “Resistant” cells were defined by the cut‐off according to serum Cmax pharmacokinetics (7.49 μM etoposide and 0.045 μM SN‐38).^20^, ^21^ H69AR and MS‐1 cells were classified as intrinsically resistant to etoposide and SN‐38. We next evaluated protein expression levels and mRNA levels of TOP1 and TOP2a (Figures 1(c) and S1). No correlation was found between sensitivity to topoisomerase inhibitors and the protein expression of TOP1 and TOP2a (*p* = 0.19, 0.35).

### Establishment of topoisomerase inhibitor‐resistant cells and identification of genes associated with resistance

**FIGURE 2:**
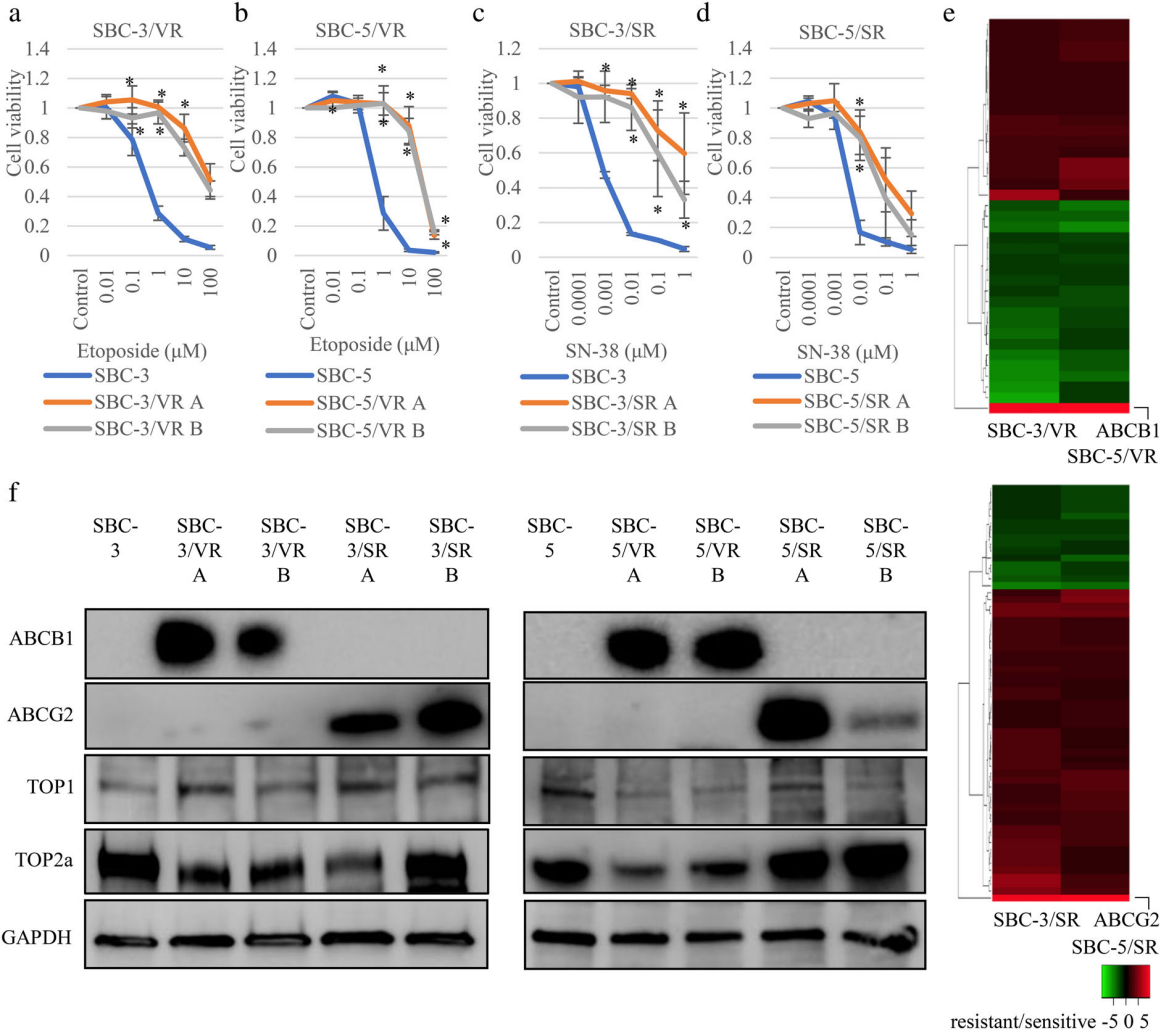
Establishment of etoposide and SN‐38 resistant small cell lung cancer (SCLC) cells. (a–d) tetrazolium (MTS) assay with topoisomerase inhibitors in established etoposide and SN‐38 resistant SCLC cells. We established two etoposide and SN‐38 resistant SBC‐3 and SBC‐5 sublines (SBC‐3/VR A, B, SBC‐5/VR A, B, SBC‐3/SR A, B, SBC‐5/SR A, B) by continuous exposure to increasing concentrations of etoposide and SN‐38. (e) RNA sequencing data (etoposide and SN‐38 resistant SCLC cells compared to sensitive parental cells). The cut‐off was over four times for resistant compared to parental sensitive lines in both SBC‐3 and SBC‐5 cell lines. Thirty‐seven and 60 genes in response to etoposide and SN‐38, respectively, were induced. ATP‐binding cassette sub‐family B member 1 (ABCB1) was overexpressed in etoposide‐resistant cells and ATP‐binding cassette sub‐family G member 2 (ABCG2) was overexpressed in SN‐38‐resistant cells compared with parental cells for both SBC‐3 and SBC‐5 cell lines (Table S1). (f) Protein expression of etoposide and SN‐38 resistant cells. Whenever the subclones resistant to etoposide showed high ABCB1 expression, the two subclones resistant to SN‐38 showed high ABCG2 expression. GAPDH, glyceraldehyde 3‐phosphate dehydrogenase; TOP, topoisomerase. *p < 0.05 compared to parental cells

To clarify the mechanism of resistance to topoisomerase inhibitors, we established SCLC cells resistant to etoposide or SN‐38. We did this using SBC‐3 and SBC‐5 cell lines that were initially sensitive to topoisomerase inhibitors but became resistant with continuous exposure to increasing concentrations of drugs for 6–8 months and subsequent subcloning. We established four etoposide‐resistant cell subclones, termed SBC‐3/VR A, B, and SBC‐5/VR A, B, using etoposide concentrations up to 4 μM, and four SN‐38‐resistant cell subclones, termed SBC‐3/SR A, B, and SBC‐5/SR A, B, using SN‐38 concentrations up to 0.04 μM (Figure 2(a)–(d)).

We next performed RNA sequencing to identify key molecules associated with resistance to etoposide and SN‐38 using two parental and four resistant cell lines. The cut‐off was over four times for resistant compared to parental sensitive lines in both SBC‐3 and SBC‐5 cell lines. Thirty‐seven and 60 genes in response to etoposide and SN‐38, respectively, were induced (Figure 2(e) and Table S2). No fusion genes expressed in both SBC‐3 and SBC‐5 resistant cell lines were found (Table S3). *RTN1, SPP1, CSMD3*, and *FST* associated with neuroendocrine features, resistance to mTOR inhibitors, resistance to etoposide in SCLC, and a diagnostic biomarker of SCLC, respectively, were included (Table S2).^9^, ^22^, ^23^, ^24^ We found ABCB1 was overexpressed in etoposide‐resistant cells and ABCG2 was overexpressed in SN‐38‐resistant cells compared with parental cells in both SBC‐3 and SBC‐5 cell lines.

We evaluated ABCB1, ABCG2, TOP1, and TOP2 protein expression in parental and resistant cells by western blotting (Figures 2(f) and S2). All subclones resistant to etoposide showed high ABCB1 expression and all resistant subclones to SN‐38 showed high ABCG2 expression. Whenever TOP2a expression was decreased in etoposide‐resistant cells, TOP1 was not decreased in SN‐38‐resistant cells. We performed MTS assays to assess the sensitivity of etoposide‐resistant cells to SN‐38 since this was also a substrate of ABCB1, as with etoposide, and showed that etoposide‐resistant cells tended to be resistant to SN‐38 (Figure S3). In addition, we performed MTS assays with topotecan, a topoisomerase inhibitor used for the treatment of SCLC, and confirmed that SN‐38‐resistant cells were resistant to topotecan (Figure S4).

### Inhibition of ABC transporters overcame resistance to topoisomerase inhibitors

**FIGURE 3:**
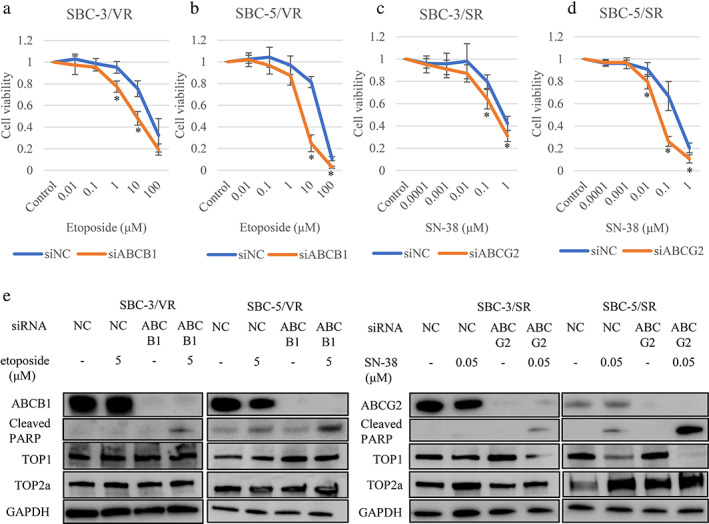
Increased sensitivity and induction of apoptosis by the inhibition of ATP‐binding cassette sub‐family B member 1 (ABCB1) or ATP‐binding cassette sub‐family G member 2 (ABCG2). (a–d) tetrazolium (MTS) assays showing ABCB1 or ABCG2 inhibition. SBC‐3/VR, SBC‐5/VR, SBC‐3/SR, and SBC‐5/SR cells following transfection with small interfering (si)RNAs displayed increased sensitivity to etoposide and SN‐38 relative to siRNA controls. (e) Protein expression in resistant cells by the inhibition of ABC transporters using siRNAs combined with topoisomerase inhibitors. The inhibition of ABC transporters by siRNA combined with topoisomerase inhibitors synergistically induced apoptosis in resistant cells. Whenever the expression of ABCB1 and ABCG2 was decreased, the expression of cleaved PARP increased. GAPDH, glyceraldehyde 3‐phosphate dehydrogenase; NC, negative control; PARP, poly ADP ribose polymerase; siABCB1, small interfering RNA to ABCB1; siABCG2, small interfering RNA to ABCG2; siNC, small interfering RNA to negative control; TOP, topoisomerase. *p < 0.05 compared to control cells

We next evaluated whether silencing ABCB1 or ABCG2 overcame the resistance to topoisomerase inhibitors. Following transfection with siRNAs, SBC‐3/VR and SBC‐5/VR cells displayed increased sensitivity to etoposide relative to siRNA controls (Figure 3(a),(b)). Following transfection with siRNAs, SBC‐3/SR and SBC‐5/SR cells also displayed a recovery of sensitivity to SN‐38 (Figure 3(c),(d)). The clonogenic response to topoisomerase inhibitors of resistant cells following transfection with siRNAs was significantly greater than that with siRNA controls (Figure S5). The inhibition of ABC transporters by siRNAs combined with topoisomerase inhibitors synergistically induced cleaved PARP expression to show apoptotic activity in resistant cells (Figures 3(e) and S6(a)–(d)). TOP1 protein levels were decreased by the inhibition of ABC transporters by siRNAs combined with topoisomerase inhibitor, but TOP2a protein levels remained unchanged (Figures 3(e) and S6(a)–(d)).

**FIGURE 4:**
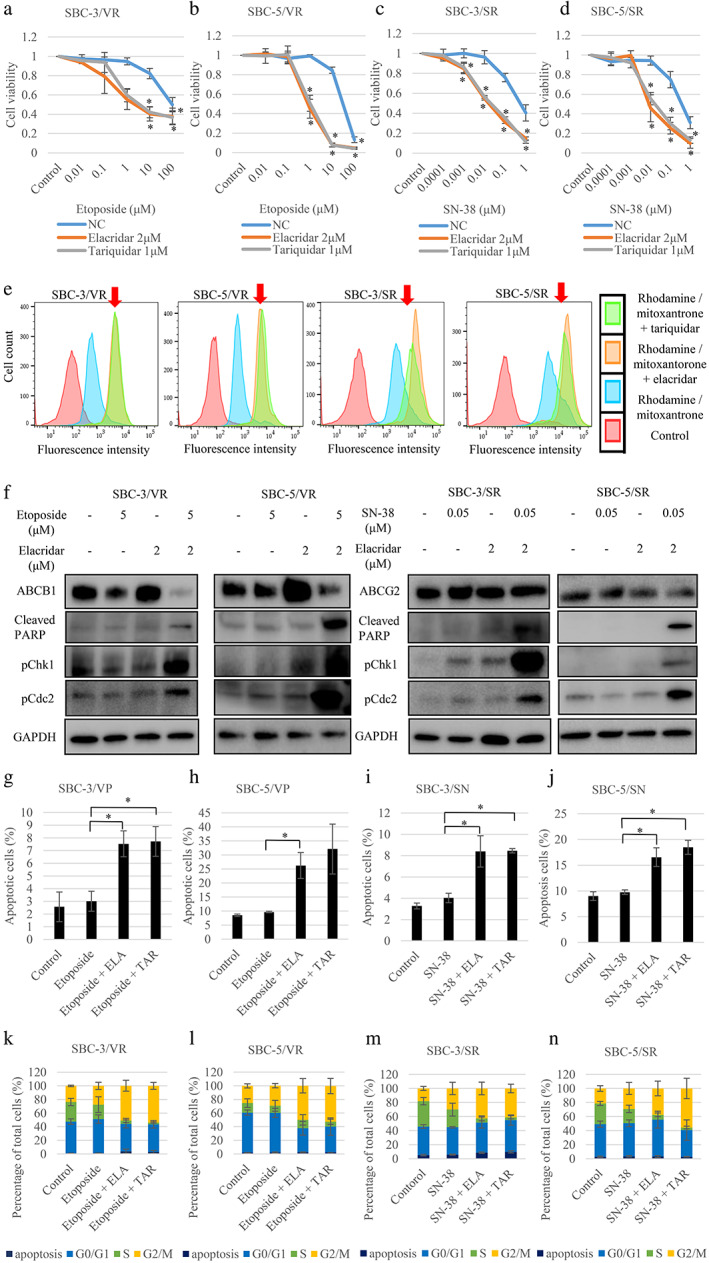
Inhibition of ABC transporters overcame resistance to topoisomerase inhibitors. (a–d) Use of the ABC transporter inhibitors elacridar and tariquidar led to a recovery of sensitivity to topoisomerase inhibitors as shown by tetrazolium (MTS) assay. (e) ABC transporter inhibitors inhibited drug efflux in topoisomerase inhibitor‐resistant cells. Transport of rhodamine123, an ATP‐binding cassette sub‐family B member 1 (ABCB1) substrate, and mitoxantrone, an ATP‐binding cassette sub‐family G member 2 (ABCG2) substrate, tended to be inhibited by elacridar and tariquidar. Histograms of untreated control cells are shown in red and cells treated with rhodamine123 or mitoxantrone are shown in blue, while orange and green indicate histograms obtained after treatment of cells with elacridar and tariquidar, respectively. A right shift in the latter compared to the blue histogram represents the inhibition of efflux through corresponding ABC transporters. (f) ABC transporter inhibitors promoted apoptosis via G2‐M arrest. The expression of proteins in resistant cells incubated with drugs was evaluated by western blot analysis. The expression of proteins associated with apoptosis (cleaved PARP) and G2‐M cell‐cycle arrest (phosphorylated‐Chk1 [p‐Chk1] and phosphorylated‐Cdc2 [p‐Cdc2]) was increased in resistant cells with etoposide or SN‐38 after treatment with elacridar. (g–j) ABC transporter inhibitors promoted apoptosis in topoisomerase inhibitor‐resistant cells. The promotion of apoptosis by drugs was evaluated by flow cytometry. Resistant cells incubated with elacridar or tariquidar and topoisomerase inhibitors promoted apoptosis. (k–n) ABC transporter inhibitors promoted G2‐M arrest in topoisomerase inhibitor‐resistant cells. The promotion of G2‐M arrest by drugs was evaluated by flow cytometry. Resistant cells incubated with elacridar or tariquidar and topoisomerase inhibitors promoted G2‐M arrest. Chk1, checkpoint kinase 1; ELA, elacridar; GAPDH, glyceraldehyde 3‐phosphate dehydrogenase; NC, normal control; PARP, poly ADP ribose polymerase; TAR, tariquidar. *p < 0.05 compared to control cells

We next examined the effect of ABCB1 and ABCG2 inhibitors. In the MTS assay, we showed recovery of sensitivity of cells to topoisomerase inhibitors with or without cisplatin by elacridar and tariquidar (Figures 4(a)–(d) and S7). We also examined the function of ABCB1 and ABCG2 inhibitors by drug efflux assay. Histograms of untreated control cells (red) and cells exposed to only rhodamine123 or mitoxantrone (blue) are shown. Orange and green indicate histograms obtained after treatment with elacridar and tariquidar, respectively. A right shift of the latter compared to the blue histogram represents the inhibition of efflux through corresponding ABC transporters. The transport of rhodamine123, an ABCB1 substrate, and mitoxantrone, an ABCG2 substrate, tended to be inhibited by elacridar and tariquidar (Figure 4(e)). After 72 h of incubation with drugs, we isolated proteins from resistant cells and evaluated protein expression by western blotting. Proteins associated with apoptosis (cleaved PARP) and G2‐M cell cycle arrest (p‐Chk1 and p‐Cdc2) were increased in cells resistant to etoposide or SN‐38 after treatment with elacridar (Figures 4(f) and S8). Flow cytometry revealed that cells cultured with ABCB1 and ABCG2 inhibitors, elacridar or tariquidar, could undergo apoptosis and G2‐M cell cycle arrest (Figures 4(g)–(n) and S9(a)–(d)). To confirm the effect of ABC transporter inhibitors, we established SBC‐3/VR and SBC‐3/SR xenograft models and treated these with or without elacridar. Combination therapy of etoposide or irinotecan and elacridar tended to inhibit tumor growth compared with vehicle and etoposide or irinotecan alone in vivo (Figure S10).

## DISCUSSION

We established etoposide‐resistant SCLC cell sublines (SBC‐3/VR and SBC‐5/VR) and SN‐38‐resistant SCLC cell sublines (SBC‐3/SR and SBC‐5/SR). Notably, *ABCB1* and *ABCG2* were the most upregulated genes in resistant cells compared with parental cells, respectively, as determined by RNA sequencing (Table S2 and Figure 2(e)). Silencing of ABCB1 and ABCG2 expression by siRNA or the inhibitors, elacridar and tariquidar, recovered the sensitivity of cells to topoisomerase inhibitors such as etoposide and SN‐38. Elacridar and tariquidar also promoted apoptosis by increased G2‐M arrest.

In RNA sequence data, ABC transporter expression was significantly higher than that of other genes, including *RTN1*, *SPP1*, *CSMD3*, and *FST*, associated with neuroendocrine features, resistance to mTOR inhibitors, resistance to etoposide in SCLC, and a diagnostic biomarker of SCLC. We therefore analyzed ABC transporters in resistant cells to reveal the relationship between sensitivity and ABC transporters. An expected resistance mechanism to topoisomerase inhibitors was in the activity or mutation of topoisomerase I.^25^, ^26^ We found the correlation between the protein expression of topoisomerase and sensitivity was small in SCLC cell lines. In resistant cell lines, high ABC transporter expression promoted topoisomerase inhibitor efflux. Whenever inhibition of ABCG2 promoted sensitivity to SN‐38 with slight TOP1 downregulation and apoptosis via G2‐M arrest, ABCB1 inhibition promoted sensitivity to etoposide without TOP2a downregulation and apoptosis via G2‐M arrest. No association was found between topoisomerase expression and sensitivity to topoisomerase inhibitor.

Other mechanisms involved in resistance to topoisomerase inhibitors were degradation of carboxylesterase and the extensive expression or mutation of efflux function.^27^ However, the resistance mechanism of SCLC is not clear. Etoposide is a substrate of ABCB1 and etoposide‐resistant cells indicated high expression of ABCB1. SN‐38 is a substrate of ABCG2 and ABCB1 but SN‐38‐resistant cells did not indicate high expression of ABCB1.

In previous studies, relationships between prognosis or multidrug resistance and protein or gene expression of ABC transporters in SCLC human tissue were investigated. According to a systematic review by Knez et al., 10 studies that included nine to 61 patients in each study described how high expression of the ABCB1 protein or gene was associated with a poor chemotherapy response rate.^28^, ^29^, ^30^, ^31^, ^32^, ^33^, ^34^, ^35^, ^36^ The largest study by Kim et al., which included 130 patients, found ABCB1 protein expression had no association with chemotherapy response rate or prognosis.^37^ With respect to relapsed patients with SCLC, Triller et al. found the ABCB1 protein expression levels of four out of five such patients increased.^28^, ^30^ Savaraj et al. described how the *ABCB1* gene level was increased in five out of seven relapsed patients with SCLC.^28^ In comparison, ABCG2 protein expression was associated with a chemotherapy response and progression‐free survival in a study by Kim et al. of 130 patients.^29^ Rijavec et al. reported in a study of 14 patients that low ABCG2 mRNA expression levels were related to longer overall survival.^38^ Whether the protein or gene expression levels of ABC transporters in patients with SCLC were associated with prognosis or multidrug resistance remained unconfirmed. It is difficult, especially in SCLC, to collect and analyze relapse or chemoresistant samples, suggesting it may be necessary to collect and analyze liquid biopsies for further investigation on SCLC human samples.

ABCB1 and ABCG2 belong to the family of ABC transporter proteins, which are energy‐dependent and normally function in the detoxification and protection of normal cells from xenobiotics.^39^ Increased ABC transporter expression is considered a significant cause of multidrug resistance to chemotherapy for various cancers.^40^, ^41^, ^42^ We have shown that ABCB1 was associated with resistance to MET inhibitors in NSCLC.^10^ ABCB1 is also associated with cancer stem cell (CSC) properties^10^; several markers, such as CD44, SOX2, and ALDH1, are possible CSC markers in SCLC.^43^ However, in our study, these markers were not upregulated in resistant cells as shown by RNA sequencing (Table S2). Although it was hypothesized ABC transporter inhibitors may recover the drug sensitivity of resistant tumors and considering many inhibitors have been developed, no clinical trials have shown the efficacy of ABC transporter inhibitors against cancers. Elacridar and tariquidar inhibited both ABCB1 and ABCG2. A phase I study demonstrated how elacridar combined with oral topotecan resulted in the complete apparent oral bioavailability of topotecan.^44^ Phase I studies of tariquidar in combination with vinorelbine, paclitaxel or doxorubicin showed no significant side effects or pharmacokinetic interactions.^45^ However, two large phase III trials of tariquidar combined with first‐line chemotherapy for patients with NSCLC closed early due to toxicity, meaning dose modification was required.^39^

In conclusion, we found that ABCB1 and ABCG2 were involved in acquired resistance to the topoisomerase inhibitors, etoposide and SN‐38, in SCLC cells. The inhibition of ABC transporters has not proven to be effective in cancers until now. However, no effective chemotherapies against recurrent SCLC currently exist and further investigations on the inhibition of ABC transporters can potentially be pivotal in the treatment patients with SCLC. Clinical trials of platinum plus etoposide or irinotecan combined with ABC transporter inhibitors in patients with SCLC are necessary.

## CONFLICT OF INTEREST

M. Seike has received a commercial research grant and honoraria from Nippon Kayaku Co., Ltd and A. Gemma received speakers' bureau honoraria from Daiichi Sankyo Co., Ltd and technical guidance fees from Nippon Kayaku Co., Ltd. The funders had no role in the design of the study; in the collection, analyses, or interpretation of data, in the writing of the manuscript, or in the decision to publish the results. The other authors have no conflicts of interest to declare.

## Supporting information


**Figure S1**. Quantification of protein expression of TOP1 (a) and TOP2a (b) on small cell lung cancer (SCLC) cell lines. There were no significant differences between sensitive and resistant cells for both TOP1/GAPDH and TOP2a/GAPDH by t‐test (p= 0.19, 0.35, respectively). The relative mRNA expression of TOP1 (c) and TOP2a (d) in the SCLC cell lines. GAPDH, glyceraldehyde 3‐phosphate dehydrogenase; TOP, topoisomerase.

**Figure S2**. Quantification of protein expression of resistant cells of SBC‐3 (a) and SBC‐5 (b). ABCB1, ATP‐binding cassette sub‐family B member 1; ABCG2, ATP‐binding cassette sub‐family G member 2; GAPDH, glyceraldehyde 3‐phosphate dehydrogenase. *p < 0.05 compared to parental cells.

**Figure S3**. Tetrazolium (MTS) assays using SBC‐3/VR A, B (a) and SBC‐5/VR A, B (b) with SN‐38. Etoposide‐resistant cells tended to be resistant to SN‐38. *p < 0.05.

**Figure S4**. Tetrazolium (MTS) assays using SBC‐3 resistant cells (a) and SBC‐5 resistant cells (b) with topotecan. SN‐38‐resistant cells were resistant to topotecan. *p < 0.05 compared to parental cells.

**Figure S5**. Colony formation assay after inhibition of ATP‐binding cassette sub‐family B member 1 (ABCB1) or ATP‐binding cassette sub‐family G member 2 (ABCG2). The clonogenic ability response to topoisomerase inhibitors of resistant cells following transfection with siRNAs was greater than that with siRNA controls. *p < 0.05.

**Figure S6**. Quantification of protein expression in resistant cells by the inhibition of ABC transporters using siRNAs combined with topoisomerase inhibitors: etoposide‐resistant cells (a, b), SN‐38‐resistant cells (c, d). ABCB1, ATP‐binding cassette sub‐family B member 1; ABCG2, ATP‐binding cassette sub‐family G member 2; GAPDH, glyceraldehyde 3‐phosphate dehydrogenase; NC, negative control; PARP, poly ADP ribose polymerase; TOP, topoisomerase. *p < 0.05.

**Figure S7**. Tetrazolium (MTS) assays using sensitive and resistant cells treated with etoposide (a, b) or SN‐38 (c, d) plus 0.2μM cisplatin. *p < 0.05 compared to resistant cells with cisplatin only.

**Figure S8**. Quantification of protein expression in resistant cells treated with topoisomerase inhibitors and elacridar: etoposide‐resistant cells with etoposide (a, b), SN‐38‐resistant cells with SN‐38 (c, d). ABCB1, ATP‐binding cassette sub‐family B member 1; ABCG2, ATP‐binding cassette sub‐family G member 2; p‐Chk1, phosphorylated‐Chk1; p‐Cdc2, phosphorylated‐Cdc2; GAPDH, glyceraldehyde 3‐phosphate dehydrogenase; PARP, poly ADP ribose polymerase. *p < 0.05.

**Figure S9**. The promotion of apoptosis was evaluated by flow cytometry: etoposide‐resistant cells (a, b), SN‐38‐resistant cells (c, d). ABC transporter inhibitors promoted apoptosis in resistant cells. ELA, elacridar; FITC, fluorescein isothiocyanate; PI, propidium iodide; TAR, tariquidar.

**Figure S10**. Combination therapy with elacridar and topoisomerase inhibitors suppresses the growth of chemoresistant tumors in vivo: SBC‐3/VR xenografts (a), SBC‐3/SR xenografts (b).

**Table S1**. RNA sequence metrics.
**Table S2**. RNA sequence data of resistant and sensitive parental cell lines.
**Table S3**. Fusion genes of resistant and sensitive parental cell lines.

## Data Availability

All data generated or analyzed during this study are included in this published article and its Supporting Information files. RNA sequencing data have been deposited in an SRA database with the accession number PRJNA717912.
