# Supplementary material for: Inhibitors of ABCB1 and ABCG2 overcame resistance to topoisomerase inhibitors in small cell lung cancer
Source: Thorac Cancer. 2022 Jun 20;13(15):2142–51. doi: 10.1111/1759-7714.14527 (PMC9346178; doi:10.1111/1759-7714.14527)
Supplement: Supplementary file 1 — Figure S1. Quantification of protein expression of TOP1 (a) and TOP2a (b) on small cell lung cancer (SCLC) cell lines. There were no significant differences between sensitive and resistant cells for both TOP1/GAPDH and TOP2a/GAPDH by t‐test (p= 0.19, 0.35, respectively). The relative mRNA expression of TOP1 (c) and TOP2a (d) in the SCLC cell lines. GAPDH, glyceraldehyde 3‐phosphate dehydrogenase; TOP, topoisomerase. [file TCA-13-2142-s011.pdf]

Figure S1.

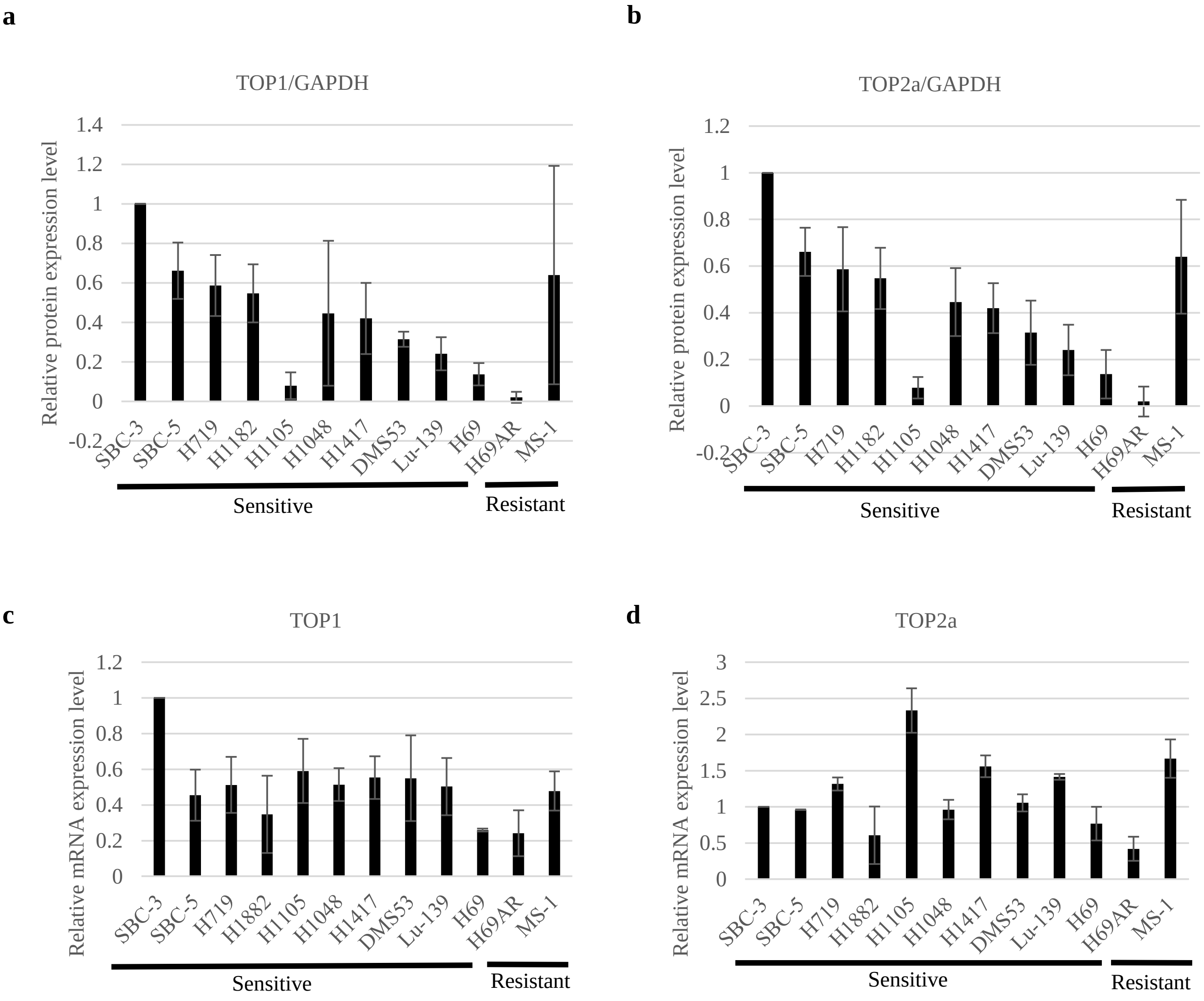

Figure S1.

Quantification of protein expression of TOP1 (a) and TOP2a (b) on small cell lung cancer (SCLC) cell lines. There were no significant differences between sensitive and resistant cells for both TOP1/GAPDH and TOP2a/GAPDH by *t*-test (p= 0.19, 0.35, respectively). The relative mRNA expression of TOP1 (c) and TOP2a (d) in the SCLC cell lines. GAPDH, glyceraldehyde 3-phosphate dehydrogenase; TOP, topoisomerase.
