# Supplementary material for: Inhibitors of ABCB1 and ABCG2 overcame resistance to topoisomerase inhibitors in small cell lung cancer
Source: Thorac Cancer. 2022 Jun 20;13(15):2142–51. doi: 10.1111/1759-7714.14527 (PMC9346178; doi:10.1111/1759-7714.14527)
Supplement: Supplementary file 2 — Figure S2. Quantification of protein expression of resistant cells of SBC‐3 (a) and SBC‐5 (b). ABCB1, ATP‐binding cassette sub‐family B member 1; ABCG2, ATP‐binding cassette sub‐family G member 2; GAPDH, glyceraldehyde 3‐phosphate dehydrogenase. *p < 0.05 compared to parental cells. [file TCA-13-2142-s002.pdf]

Figure S2.

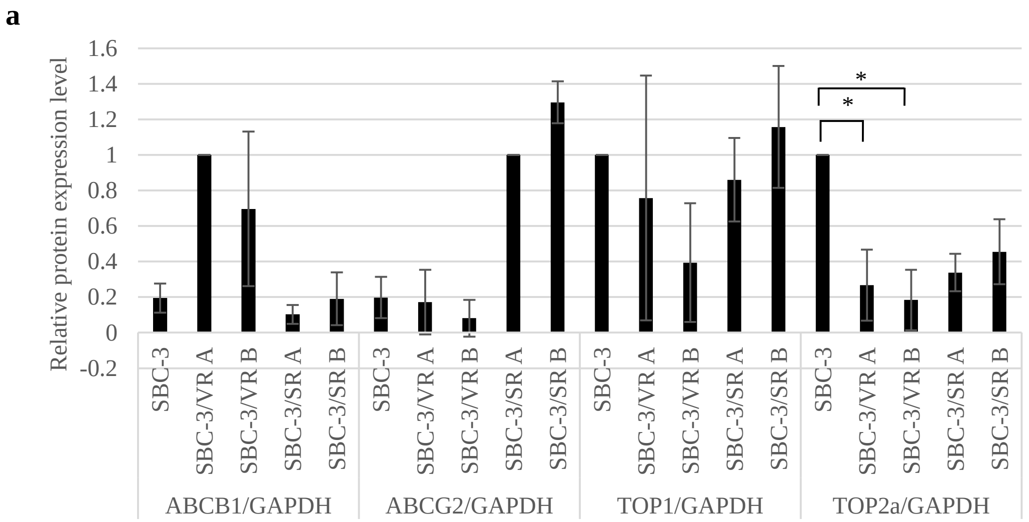

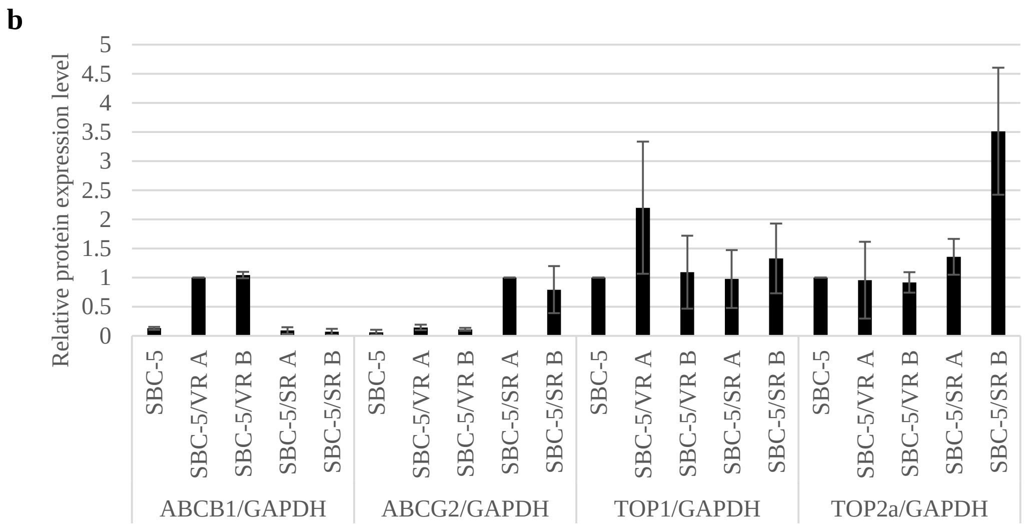

Figure S2.

Quantification of protein expression of resistant cells of SBC-3 (a) and SBC-5 (b). ABCB1, ATP-binding cassette sub-family B member 1; ABCG2, ATP-binding cassette sub-family G member 2; GAPDH, glyceraldehyde 3-phosphate dehydrogenase. *$p < 0.05$
