# Supplementary material for: Inhibitors of ABCB1 and ABCG2 overcame resistance to topoisomerase inhibitors in small cell lung cancer
Source: Thorac Cancer. 2022 Jun 20;13(15):2142–51. doi: 10.1111/1759-7714.14527 (PMC9346178; doi:10.1111/1759-7714.14527)
Supplement: Supplementary file 3 — Figure S3. Tetrazolium (MTS) assays using SBC‐3/VR A, B (a) and SBC‐5/VR A, B (b) with SN‐38. Etoposide‐resistant cells tended to be resistant to SN‐38. *p < 0.05. [file TCA-13-2142-s008.pdf]

Figure S3.

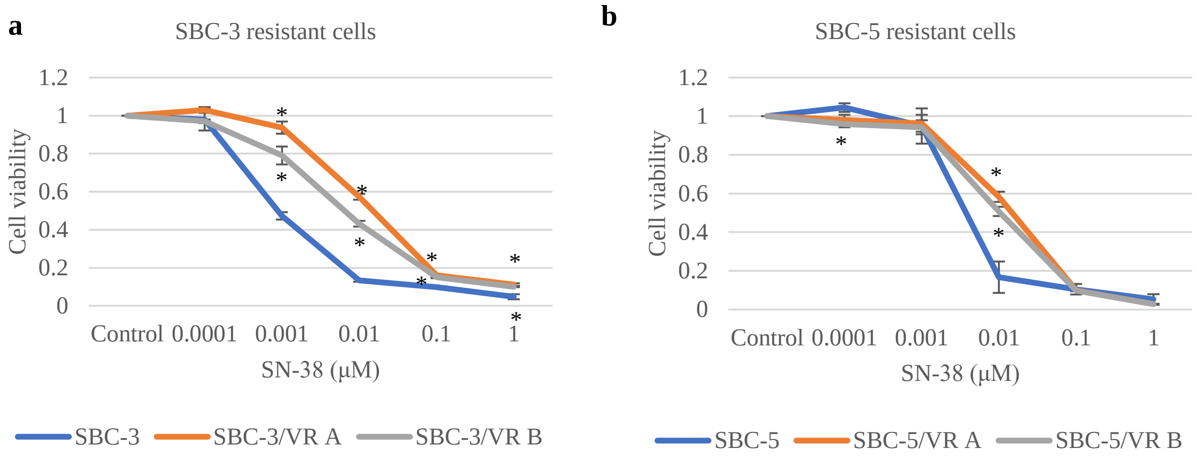

Figure S3.

Tetrazolium (MTS) assays using SBC-3/VR A, B (a) and SBC-5/VR A, B (b) with SN-38. Etoposide-resistant cells tended to be resistant to SN-38. **p* < 0.05 compared to parental cells.
