# Supplementary material for: Inhibitors of ABCB1 and ABCG2 overcame resistance to topoisomerase inhibitors in small cell lung cancer
Source: Thorac Cancer. 2022 Jun 20;13(15):2142–51. doi: 10.1111/1759-7714.14527 (PMC9346178; doi:10.1111/1759-7714.14527)
Supplement: Supplementary file 4 — Figure S4. Tetrazolium (MTS) assays using SBC‐3 resistant cells (a) and SBC‐5 resistant cells (b) with topotecan. SN‐38‐resistant cells were resistant to topotecan. *p < 0.05 compared to parental cells. [file TCA-13-2142-s001.pdf]

Figure S4.

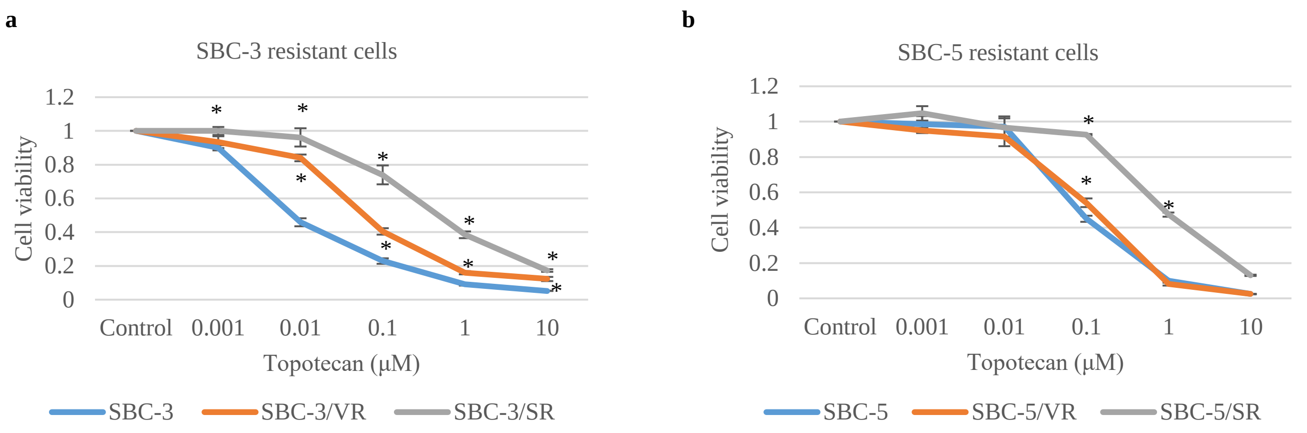

Figure S4.

Tetrazolium (MTS) assays using SBC-3 resistant cells (a) and SBC-5 resistant cells (b) with topotecan. SN-38-resistant cells were resistant to topotecan. *$p < 0.05$ compared to parental cells.
