# Supplementary material for: Inhibitors of ABCB1 and ABCG2 overcame resistance to topoisomerase inhibitors in small cell lung cancer
Source: Thorac Cancer. 2022 Jun 20;13(15):2142–51. doi: 10.1111/1759-7714.14527 (PMC9346178; doi:10.1111/1759-7714.14527)
Supplement: Supplementary file 5 — Figure S5. Colony formation assay after inhibition of ATP‐binding cassette sub‐family B member 1 (ABCB1) or ATP‐binding cassette sub‐family G member 2 (ABCG2). The clonogenic ability response to topoisomerase inhibitors of resistant cells following transfection with siRNAs was greater than that with siRNA controls. *p < 0.05. [file TCA-13-2142-s006.pdf]

Figure S5.

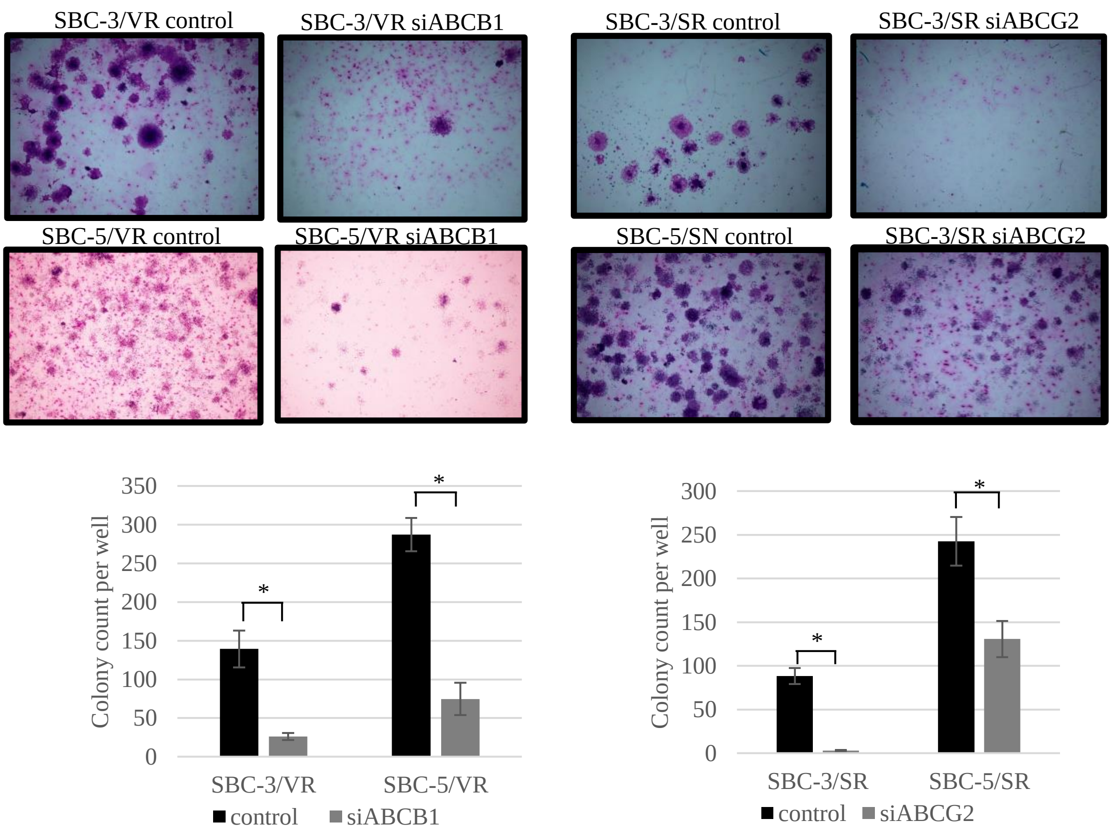

Figure S5.

Colony formation assay after inhibition of ATP-binding cassette sub-family B member 1 (ABCB1) or ATP-binding cassette sub-family G member 2 (ABCG2). The clonogenic ability response to topoisomerase inhibitors of resistant cells following transfection with siRNAs was greater than that with siRNA controls. *$p < 0.05$
