# Supplementary material for: Inhibitors of ABCB1 and ABCG2 overcame resistance to topoisomerase inhibitors in small cell lung cancer
Source: Thorac Cancer. 2022 Jun 20;13(15):2142–51. doi: 10.1111/1759-7714.14527 (PMC9346178; doi:10.1111/1759-7714.14527)
Supplement: Supplementary file 6 — Figure S6. Quantification of protein expression in resistant cells by the inhibition of ABC transporters using siRNAs combined with topoisomerase inhibitors: etoposide‐resistant cells (a, b), SN‐38‐resistant cells (c, d). ABCB1, ATP‐binding cassette sub‐family B member 1; ABCG2, ATP‐binding cassette sub‐family G member 2; GAPDH, glyceraldehyde 3‐phosphate dehydrogenase; NC, negative control; PARP, poly ADP ribose polymerase; TOP, topoisomerase. *p < 0.05. [file TCA-13-2142-s005.pdf]

Figure S6.

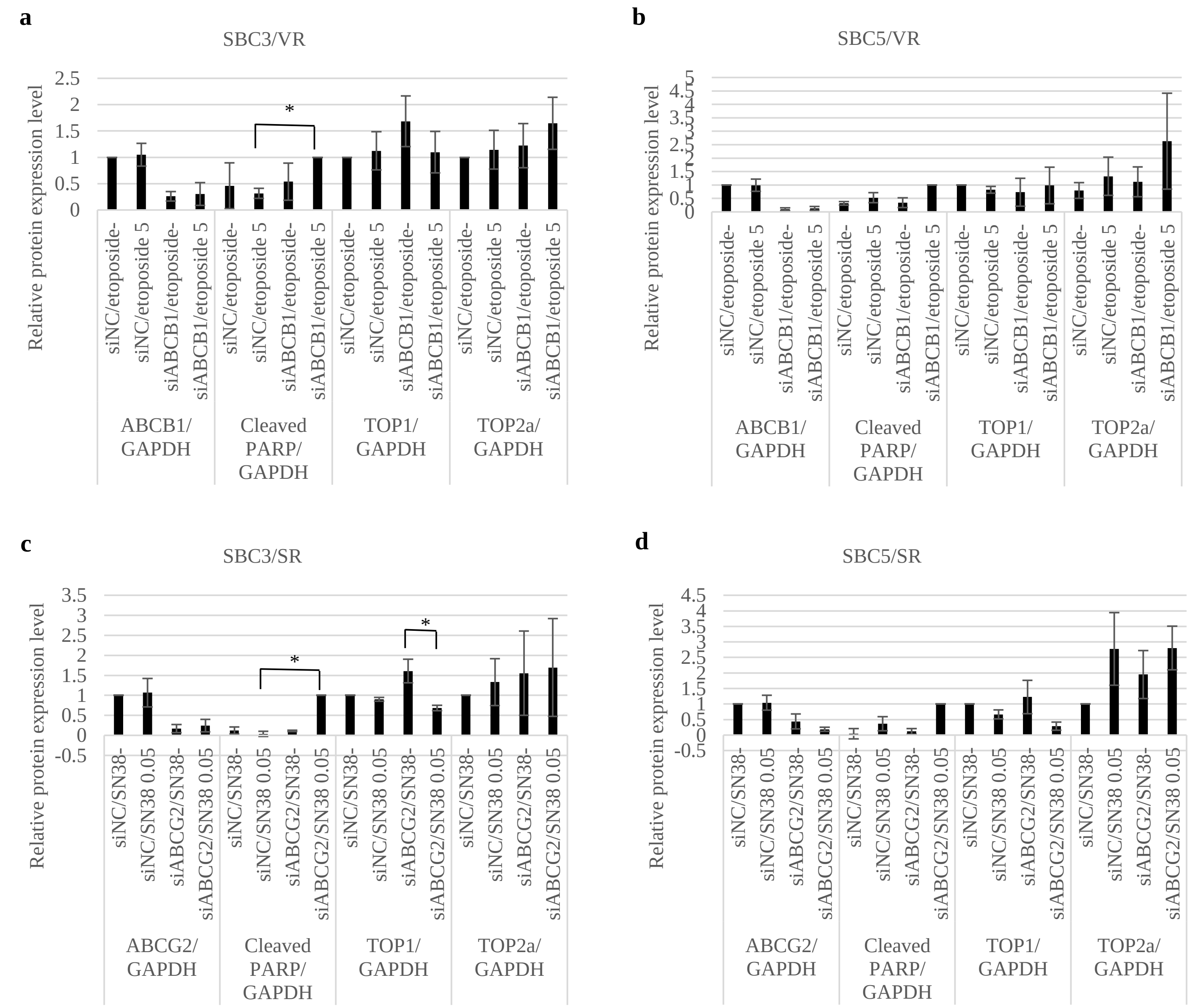

Figure S6.

Quantification of protein expression in resistant cells by the inhibition of ABC transporters using siRNAs combined with topoisomerase inhibitors: etoposide-resistant cells (a, b), SN-38–resistant cells (c, d). ABCB1, ATP-binding cassette sub-family B member 1; ABCG2, ATP-binding cassette sub-family G member 2; GAPDH, glyceraldehyde 3-phosphate dehydrogenase; NC, negative control; PARP, poly ADP ribose polymerase; TOP, topoisomerase. *p < 0.05
