# Supplementary material for: Inhibitors of ABCB1 and ABCG2 overcame resistance to topoisomerase inhibitors in small cell lung cancer
Source: Thorac Cancer. 2022 Jun 20;13(15):2142–51. doi: 10.1111/1759-7714.14527 (PMC9346178; doi:10.1111/1759-7714.14527)
Supplement: Supplementary file 7 — Figure S7. Tetrazolium (MTS) assays using sensitive and resistant cells treated with etoposide (a, b) or SN‐38 (c, d) plus 0.2μM cisplatin. *p < 0.05 compared to resistant cells with cisplatin only. [file TCA-13-2142-s007.pdf]

Figure S7.

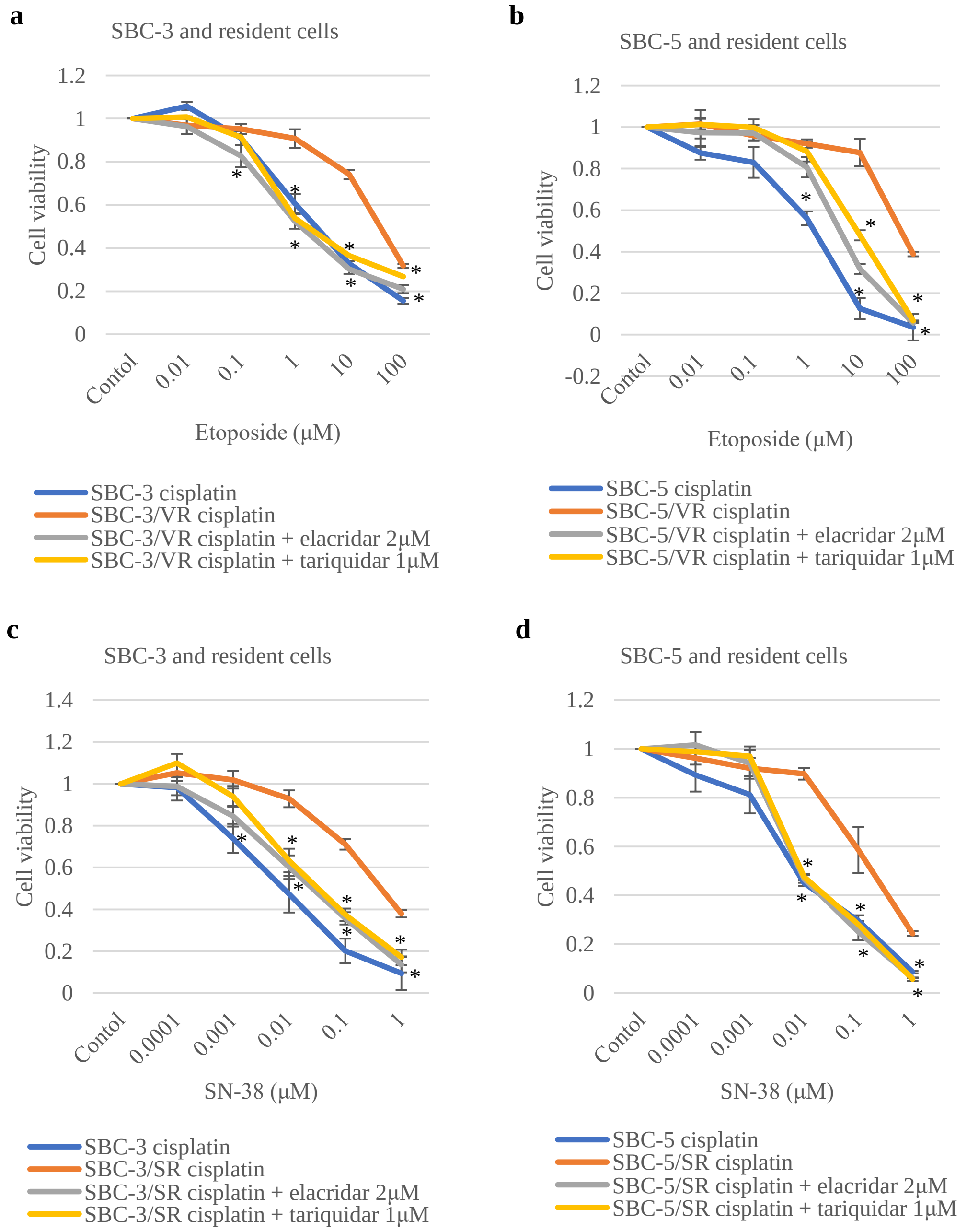

Figure S7.

Tetrazolium (MTS) assays using sensitive and resistant cells treated with etoposide (a, b) or SN-38 (c, d) plus 0.2µM cisplatin. *$p < 0.05$ compared to resistant cells with cisplatin only.
