# Supplementary material for: Inhibitors of ABCB1 and ABCG2 overcame resistance to topoisomerase inhibitors in small cell lung cancer
Source: Thorac Cancer. 2022 Jun 20;13(15):2142–51. doi: 10.1111/1759-7714.14527 (PMC9346178; doi:10.1111/1759-7714.14527)
Supplement: Supplementary file 8 — Figure S8. Quantification of protein expression in resistant cells treated with topoisomerase inhibitors and elacridar: etoposide‐resistant cells with etoposide (a, b), SN‐38‐resistant cells with SN‐38 (c, d). ABCB1, ATP‐binding cassette sub‐family B member 1; ABCG2, ATP‐binding cassette sub‐family G member 2; p‐Chk1, phosphorylated‐Chk1; p‐Cdc2, phosphorylated‐Cdc2; GAPDH, glyceraldehyde 3‐phosphate dehydrogenase; PARP, poly ADP ribose polymerase. *p < 0.05. [file TCA-13-2142-s004.pdf]

Figure S8.

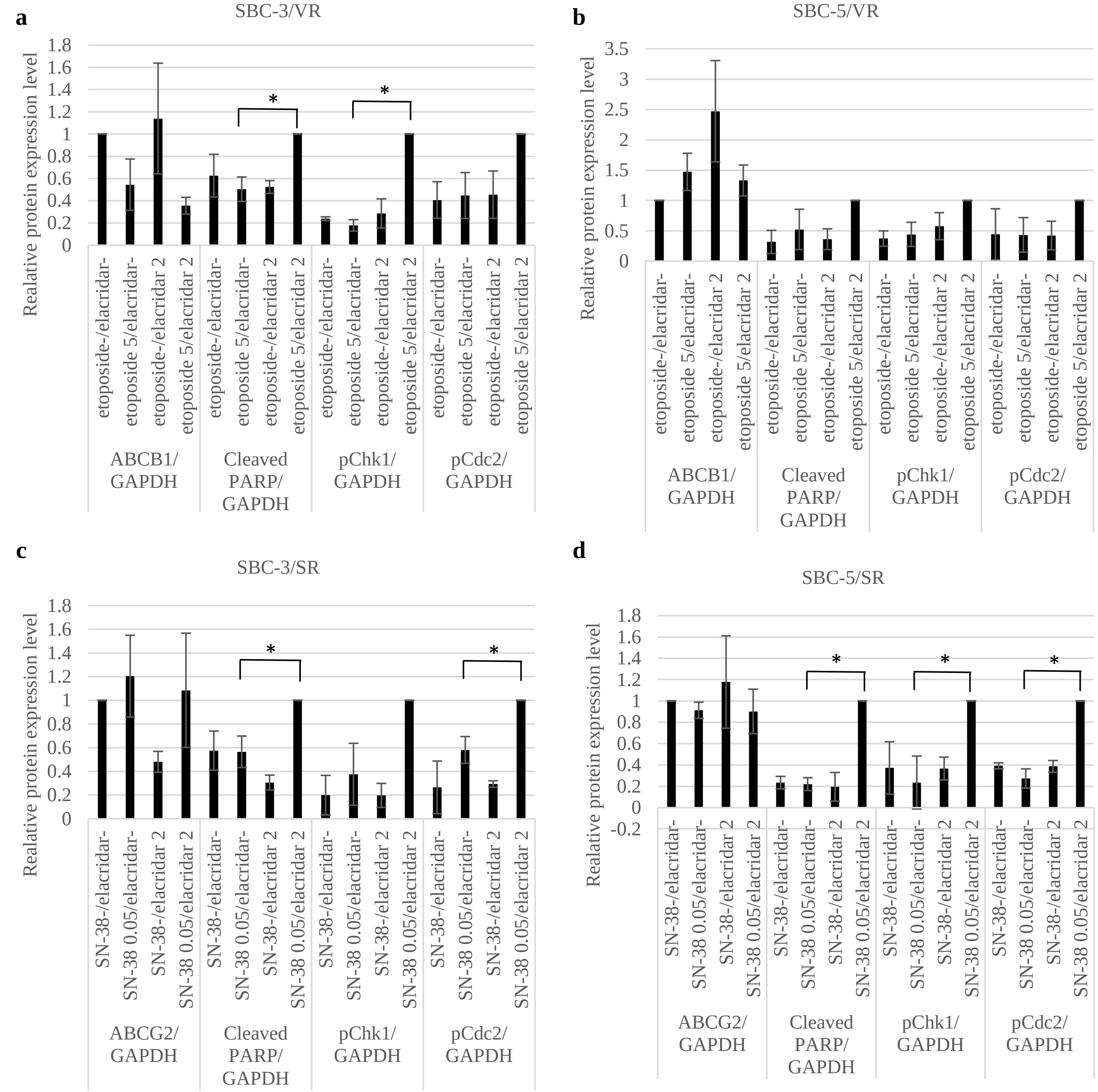

Figure S8.

Quantification of protein expression in resistant cells treated with topoisomerase inhibitors and elacridar: etoposide-resistant cells with etoposide (a, b), SN-38–resistant cells with SN-38 (c, d). ABCB1, ATP-binding cassette sub-family B member 1; ABCG2, ATP-binding cassette sub-family G member 2; p-Chk1, phosphorylated-Chk1; p-Cdc2, phosphorylated-Cdc2; GAPDH, glyceraldehyde 3-phosphate dehydrogenase; PARP, poly ADP ribose polymerase. *$p < 0.05$
