# Supplementary material for: Inhibitors of ABCB1 and ABCG2 overcame resistance to topoisomerase inhibitors in small cell lung cancer
Source: Thorac Cancer. 2022 Jun 20;13(15):2142–51. doi: 10.1111/1759-7714.14527 (PMC9346178; doi:10.1111/1759-7714.14527)
Supplement: Supplementary file 9 — Figure S9. The promotion of apoptosis was evaluated by flow cytometry: etoposide‐resistant cells (a, b), SN‐38‐resistant cells (c, d). ABC transporter inhibitors promoted apoptosis in resistant cells. ELA, elacridar; FITC, fluorescein isothiocyanate; PI, propidium iodide; TAR, tariquidar. [file TCA-13-2142-s003.pdf]

Figure S9.

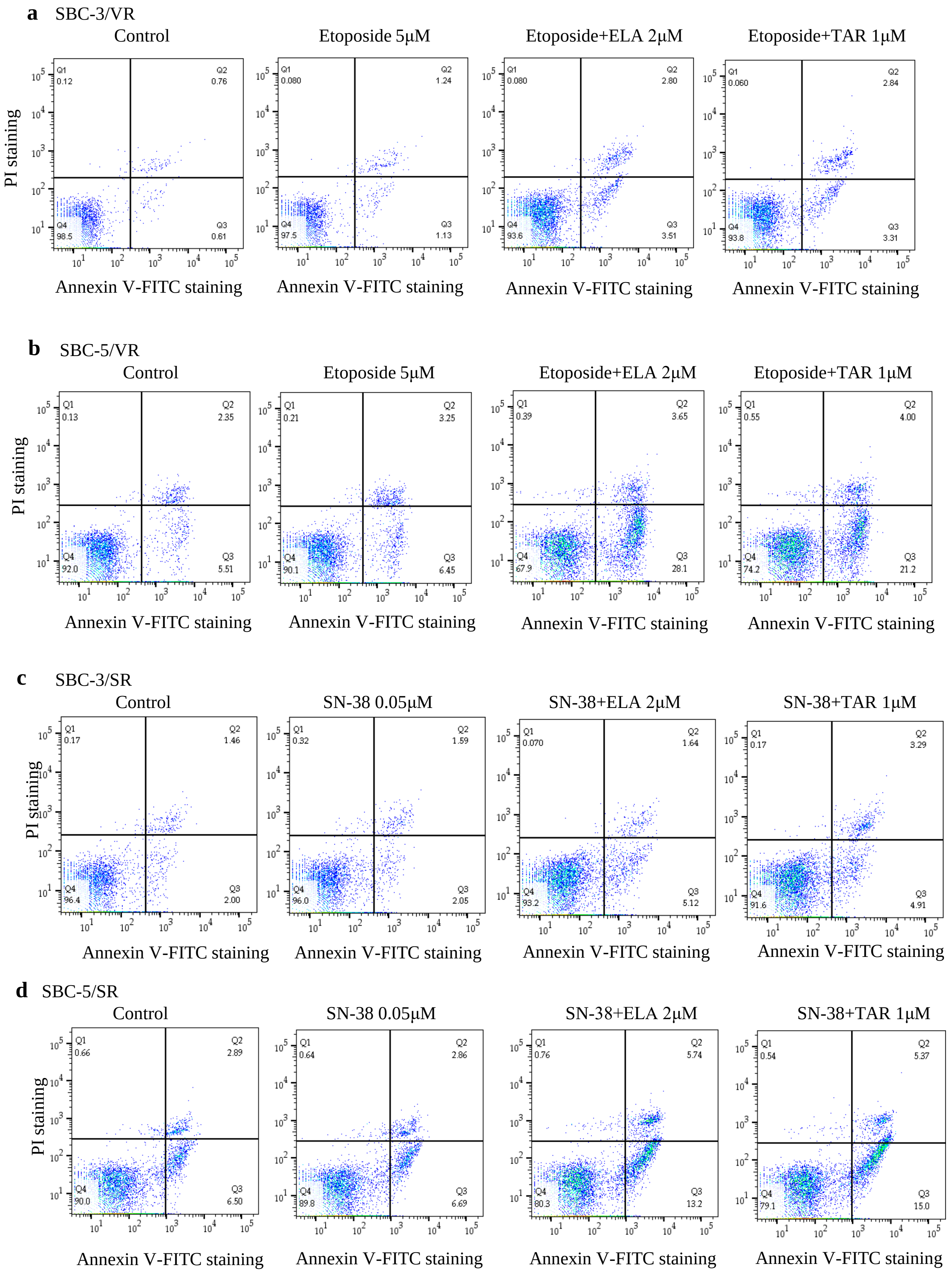

Figure S9.

The promotion of apoptosis was evaluated by flow cytometry: etoposide-resistant cells (a, b), SN-38–resistant cells (c, d). ABC transporter inhibitors promoted apoptosis in resistant cells. ELA, elacridar; FITC, fluorescein isothiocyanate; PI, propidium iodide; TAR, tariquidar.
