# Supplementary material for: Inhibitors of ABCB1 and ABCG2 overcame resistance to topoisomerase inhibitors in small cell lung cancer
Source: Thorac Cancer. 2022 Jun 20;13(15):2142–51. doi: 10.1111/1759-7714.14527 (PMC9346178; doi:10.1111/1759-7714.14527)
Supplement: Supplementary file 10 — Figure S10. Combination therapy with elacridar and topoisomerase inhibitors suppresses the growth of chemoresistant tumors in vivo: SBC‐3/VR xenografts (a), SBC‐3/SR xenografts (b). [file TCA-13-2142-s009.pdf]

Figure S10.

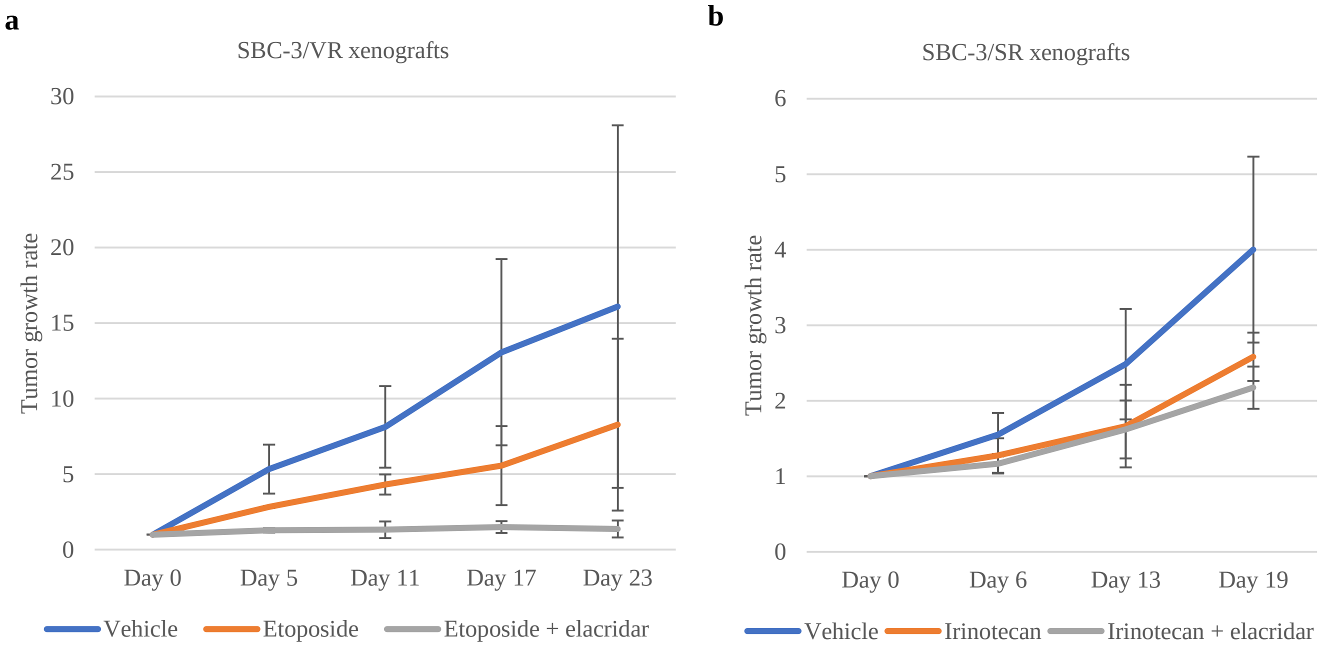

Figure S10.

Combination therapy with elacridar and topoisomerase inhibitors suppresses the growth of chemoresistant tumors *in vivo*: SBC-3/VR xenografts (a), SBC-3/SR xenografts (b).
